# Supplementary material for: Quantifying dynamic facial expressions under naturalistic conditions
Source: eLife. 2022 Aug 31;11:e79581. doi: 10.7554/eLife.79581 (PMC9439684; doi:10.7554/eLife.79581)
Supplement: Supplementary file 1. [file elife-79581-supp1.doc]

**Supplementary File 1**

| **Short description** | **Detailed description** | **Start time (seconds)** |
| --- | --- | --- |
| Happiness | Cute dog saying “I love you”  Young girl on singing show | 0 |
| Surprise | Angry office worker breaks telephone  Man surprised by loud bang | 40 |
| Disgust | Man eats beetle larva | 70 |
| Fear | Large snake swallows a pig | 96 |
| Sadness | Starvation, funerals | 125 |
| Fear | Crocodile show ends with crocodile snapping on man’s hand | 199 |

Supplementary file 1a. Videos shown to participants in the DISFA dataset.

| Action unit | Description | Associated emotion |
| --- | --- | --- |
| **1** | Inner Brow Raiser | Sadness, surprise, fear |
| **4** | Brow Lowerer | Sadness, fear, anger |
| **6** | Cheek Raiser | Happiness |
| **7** | Lid Tightener | Fear, anger |
| **9** | Nose Wrinkler | Disgust |
| **10** | Upper Lip Raiser |  |
| **12** | Lip Corner Puller | Happiness |
| **14** | Dimpler |  |
| **15** | Lip Corner Depressor | Sadness, disgust |
| **17** | Chin Raiser | Disgust |
| **20** | Lip Stretcher | Fear |
| **23** | Lip Tightener | Anger |
| **25** | Lips Part |  |
| **26** | Jaw Drop | Surprise, fear |

Supplementary file 1b. Facial action units used in our study, with corresponding time series extracted with OpenFace. The third column shows emotions conventionally associated with each action unit, in the Emotional Facial Action Coding System.

| **Model** | **Number of input features** | **Individual trial accuracies** | **Mean accuracy** |
| --- | --- | --- | --- |
| Model 1: Time-frequency representation in 10 frequency bands | 420 | 73%, 70%, 75%, 67%, 71% | 71% |
| Model 2: Mean activation | 42 | 64% 67%, 67%, 60%, 59% | 63%* |
| Model 3A: Mean activation in 30s time chunks | 224 | 64%, 64%, 67%, 60%, 65% | 64%* |
| Model 3B: Mean activation in 10s time chunks | 462 | 67%, 62%, 64%, 59%, 62% | 63%* |
| Model 3C: Mean activation in 2s time chunks | 2464 | 59%, 60%, 68%, 67%, 64% | 64%* |

Supplementary file 1c. Models to classify participants with melancholia from healthy controls. All models used support vector machine with Gaussian kernel, and were tested with 5-fold cross validation.

*p<0.05 for difference in classification loss compared to Model 1

| **Parameter** | **Description** | **Value** |
| --- | --- | --- |
| K | Maximum number of HMM states | 8 |
| Order | Maximum order of multivariate auto-regressive model. 0 for no auto-regression. | 0 |
| DirichletDiag | Value of the diagonal of the prior of the transition probability matrix | 1 |
| pca | Number of top PCA components used | 10 |
| downsample | New sampling rate (Hz) | 10 |
| cyc | Maximum number of variational inference cycles | 500 |
| initcyc | Number of repetitions of the initialisation algorithm | 10 |

Supplementary file 1d. Parameters for HMM implemented in the HMM-MAR toolbox. Parameters not listed here are left to default options.
